# Association between subfield volumes of the medial temporal lobe and cognitive assessments

**DOI:** 10.1016/j.heliyon.2019.e01828

**Published:** 2019-06-04

**Authors:** Masayo Ogawa, Daichi Sone, Iman Beheshti, Norihide Maikusa, Kyoji Okita, Harumasa Takano, Hiroshi Matsuda

**Affiliations:** Integrative Brain Imaging Center, National Center of Neurology and Psychiatry, Kodaira, Tokyo, Japan

**Keywords:** Medical imaging, Alzheimer's disease, Mild cognitive impairment, Magnetic resonance imaging, Hippocampal subfields, Cognitive assessments

## Abstract

Cognitive assessments and neuroimaging are routinely combined in clinical practice to diagnose dementia represented by Alzheimer's disease (AD). The Montreal Cognitive Assessment (MoCA) is reported to be more suitable than the Mini-Mental State Examination (MMSE) for screening mild cognitive impairment (MCI) and mild AD. On the other hand, attention to the subfield volumes of the medial temporal lobe has recently been considered important for the differential diagnosis and early detection of AD. The aim of this study was to uncover which specific hippocampal subfields and adjacent extrahippocampal structures contribute to deficits in cognitive assessment scores in patients with MCI and AD. We recruited from our institute 31 Japanese patients—14 with amnestic MCI and 17 with probable AD, with a clinical dementia rating (CDR) of 0.5 and 1, respectively—and 50 healthy elderly individuals with a CDR of 0. All participants underwent magnetic resonance imaging and cognitive assessments with the MMSE, Wechsler Memory Scale-Revised Logical Memory I and II, and Japanese version of the MoCA (MoCA-J). With adjustment for age and sex, we performed partial correlation analysis of the cognitive assessment scores with the subfield volumes of the medial temporal lobe measured by software-mediated automatic segmentation of hippocampal subfields using high-resolution T1-and T2-weighted images. Compared with normal controls, patients with MCI and AD showed subfield volume reductions in cornu ammonis (CA) 1, CA2, Brodmann area (BA) 35, BA36, the dentate gyrus (DG), the subiculum, and the entorhinal cortex (ERC). All participants showed high correlation coefficients (above 0.6) between cognitive assessment scores and subfield volumes in CA1, the DG, the subiculum, the ERC, and BA36. In patients with MCI and AD, the MoCA-J showed higher correlations than the MMSE with subfield volumes in CA1, the DG, the subiculum, and the ERC. These results suggest that the combination of the in vivo analysis of subfield morphometry of the medial temporal lobe with the MoCA-J paradigm provides important insights into whether changes within specific subfields are related to the cognitive profile in MCI and AD.

## 1 Introduction

According to the World Health Organization, the proportion of the population over the age of 60 years is increasing year by year, with the total number of dementia patients projected to increase to 82 million in 2030 and to 152 million in 2050 [1]. In dementia represented by Alzheimer's disease (AD), early detection allows for rapid assessment and treatment of reversible or treatable causes [2]. Accurate and early diagnosis plays an important role in patient care and the development of future treatments [2, 3]. Cognitive impairment is a serious health problem that undermines the active independent living of the elderly and ultimately threatens survival. Thus, it is important to screen older persons with mild cognitive impairment (MCI), the prodromal stage of AD, and to provide them with an appropriate intervention.

Cognitive assessments help physicians to assess cognitive function in individuals manifesting cognitive impairment. Among different cognitive assessments, the Mini-Mental State Examination (MMSE) has been widely used and can be easily implemented in clinical practice [4]. However, as a drawback, the MMSE has often been criticized for its poor screening sensitivity for mild dementia and MCI [5, 6]. Therefore, the Montreal Cognitive Assessment (MoCA) was developed in Canada to screen patients falling within the normal range in the MMSE [7]. As of 2017, the MoCA has been translated into 46 languages. In Japan, Fujiwara et al. [8] confirmed the reliability and validity of the Japanese version (MoCA-J). Although the MoCA is aimed at MCI screening, it is also suitable for mild AD screening [7].

Magnetic resonance imaging (MRI) is useful for objective and noninvasive evaluation of specific brain atrophy for AD. AD shows selective atrophy in the medial temporal lobe including the hippocampus and parahippocampal areas. A reduced hippocampal volume is associated with cognitive impairment in patients with AD and may serve as a prognostic neuroimaging biomarker of early cognitive impairment [9]. The hippocampus, the centerpiece of the medial temporal lobe, can be divided into subfields such as the cornu ammonis (CA1, CA2, CA3, and CA4), dentate gyrus (DG), and subiculum. Selective atrophy of the entorhinal cortex (ERC), perirhinal cortex, subiculum, and CA1 has been reported in the early stage of AD [10, 11, 12]. Furthermore, a correlation between tau or amyloid deposits and the subfield volumes of the medial temporal lobe has also been reported in mild AD [13]. Accordingly, attention to the subfield volumes of the medial temporal lobe is considered important for the differential diagnosis and early detection of AD.

Cognitive assessments and neuroimaging are routinely combined in clinical practice to diagnose dementia. Clarification of the neuroimaging findings related to cognitive function due to normal aging, MCI, and AD may lead to the early diagnosis of AD. Accordingly, several investigations have been performed to study patients with AD by combining volumetric assessment of the entire hippocampus and its subfields with clinical assessment of Cognitive tests. Lim et al. [14] found that the volume of the left and right subiculum is correlated with the Korean version of the MMSE. Novellino et al. [15] demonstrated a positive correlation between the Free and Cued Selective Reminding Test (FCSRT) and the CA4+DG volume. The FCSRT measures only one aspect of the cognitive function of word memory. The lengthy combination of several test batteries places considerable burden on the elderly and is not suitable for routine clinical studies. Meanwhile, the MoCA is a cognitive assessment that can measure multifaceted cognitive function composed of execution function, visual space recognition, memory, and other aspects and can be quickly implemented. The present study was designed to investigate which specific hippocampal subfields and adjacent extrahippocampal structures contribute to deficits in cognitive assessment scores (including MoCA-J) in patients with MCI and AD.

## 2 Materials and methods

### 2.1 Participant characteristics

Demographic data of participants are shown in Table1. We recruited from our institute 31 Japanese patients (19 women, 12 men)—14 with amnestic MCI and 17 with probable AD, with a clinical dementia rating (CDR) [16, 17] of 0.5 and 1, respectively—and 50 healthy elderly participants (27 women, 23 men) with a CDR of 0. Patients were diagnosed based on the clinical criteria of the National Institute on Aging and the Alzheimer's Association for MCI due to AD [18] and dementia due to AD [19]. All participants underwent MRI and cognitive assessments with the MMSE, Wechsler Memory Scale-revised Logical Memory I and II (WMS-R LM I and II) [20], and MoCA-J. Those with psychiatric and neurological diseases, cardiovascular diseases, pacemakers or metals in the body, and claustrophobia were excluded. All clinical assessments and MRI were performed within a 12-week period.

**Table 1:** Demographic and clinical features of healthy elderly controls and patients with MCI and AD.

	HC (n = 50)	MCI (n = 14)	AD (n = 17)	F	*p* value intergroups
	Mean ± S.D.	Mean ± S.D.	mean ± S.D.		
age	66.32 ± 8.5	70.3 ± 10.1	71.9 ± 9.1	n.s	―
M/f	23:27	6:8	6:11	n.s	―
education years	14.1 ± 2.6	13.3 ± 2.5	13.1 ± 2.9	n.s	―
MMSE	29.0 ± 1.4	26.3 ± 4.0	21.4 ± 2.4	72.875∗	HC×MCI: p < 0.001∗
					HC×AD: p < 0.001∗
					MCI×AD: p < 0.001∗
MoCA-J	27.2 ± 2.3	21.9 ± 5.6	17.4 ± 2.9	64.328∗	HC×MCI: p < 0.001∗
					HC×AD: p < 0.001∗
					MCI×AD: p = 0.001∗
WMS-R LMⅠ	14.7 ± 3.5	9.0 ± 4.8	5.7 ± 3.2	43.389∗	HC×MCI: p < 0.001∗
					HC×AD: p < 0.001∗
					MCI×AD: p = 0.037∗
WMS-R LMⅡ	12.7 ± 3.4	6.8 ± 5.4	2.3 ± 3.3	50.989∗	HC×MCI: p < 0.001∗
					HC×AD: p < 0.001∗
					MCI×AD: p = 0.005∗

AD, Alzheimer's disease; CDR, Clinical Dementia Rating; F, female; HC, healthy control; M, male; MCI, mild cognitive impairment; MMSE, Mini-Mental State Examination; MoCA-J, Montreal Cognitive Assessment Japanese Version; ns, not significant; WMS-R LM I, Wechsler Memory Scale-revised Logical Memory I, WMS-R LM II; Wechsler Memory Scale-revised Logical Memory II.

Post hoc comparison: Bonferroni.

∗ *p* < 0.05.

### 2.2 Image acquisition

MRI scanning was performed using a 3.0-T MRI system (Verio, Siemens, Erlangen, Germany). Three-dimensional (3D) sagittal T1-weighted magnetization prepared rapid acquisition with gradient echo (MPRAGE) images were obtained as follows: repetition time/echo time, 1900 ms/2.52 ms; flip angle, 9°; in-plane resolution, 1.0 × 1.0 mm; 1.0-mm effective slice thickness with no gap; 300 slices; matrix, 256 × 256; field of view, 25 × 25 cm; acquisition time, 4 mins 18 s.

High-resolution T2-weighted images were designed for hippocampal subfield segmentation and obtained as follows: in-plane resolution, repetition time/echo time, 7380 ms/76 ms; flip angle, 150°; in-plane resolution, 0.4 × 0.4 mm; 2-mm slice thickness with no gap; 30 slices; matrix of 512 × 432; 22 × 22 cm field of view; acquisition time; 6 min 33 s.

Radiologic interpretations on quality control and gross organic lesions were performed by a neuroradiologist (HM).

### 2.3 Subfield volumetry of the medial temporal lobe

We measured subfield volumes using automatic segmentation of hippocampal subfields (ASHS) software developed at the University of Pennsylvania [21, 22]. The ASHS segments hippocampal subfields on the basis of a multi-atlas segmentation with joint label fusion, and a bias correction using advanced machine-learning techniques. To achieve the optimal segmentation, ASHS uses both high-resolution T1-weighted and T2-weighted images. The main advantage of ASHS segmentation software is that it is a fully automated framework at all stages (i.e., MRI pre-processing, image segmentation, bias correction and refining), which parcellates medial temporal lobe into the following subfields: CA1, CA2, CA3, the DG, the subiculum, the ERC, the perirhinal cortex of Brodmann area (BA) 35 and BA36, the collateral sulcus, and a miscellaneous part (MISC). The technical details of ASHS segmentation software as well as the comparison between hippocampal subfield segmentation by ASHS and manual approach is described elsewhere [22]. The ASHS segmentation software is available at https://sites.google.com/site/hipposubfields/. To suppress variations in individual head sizes, all subfield volumes of the medial temporal lobe were adjusted by the total intracranial volume.

Moreover a previous study has suggested that ASHS may have advantages in compatibility with existing histopathologic knowledge compared to FreeSurfer ver. 5.3 [23].

### 2.4 Statistical analysis

The differences in age, years of education, and cognitive assessment score (i.e., MMSE, MoCA-J, WMS-R LM I, and WMS-R LM II) among the three groups were evaluated by analysis of variance (ANOVA), Welch's test if not equal variance and a post-hoc comparisons Bonferroni for intergroup. Sex differences among the three groups were evaluated by the chi-square test. Partial correlation analysis between subfield volumes of the medial temporal lobe and the cognitive assessment scores was performed with adjustment for age and sex using SPSS software (version 23.0; IBM Japan, Tokyo, Japan).

A *p* value <0.05 was considered significant.

### 2.5 Ethical considerations

All individuals gave written informed consent to participate in the study. This study was approved by the ethics committee of the National Center of Neurology and Psychiatry (A2014–146,A2014-058).

## 3 Results

### 3.1 Clinical findings

The demographic and clinical characteristics of the participants are shown in Table 1. There were no significant differences in age, years of education, and sex among the three diagnostic groups. There were significant differences in MMSE, MoCA-J, and WMS-R LM I and II scores among the three groups (*p* < 0.05). Post hoc comparisons with Bonferroni showed significant differences between all groups in MMSE, MoCA-J, and WMS-R LM I and II(*p* < 0.05).

### 3.2 Subfield volumes of the medial temporal lobe measured by ASHS

**Fig. 1:**
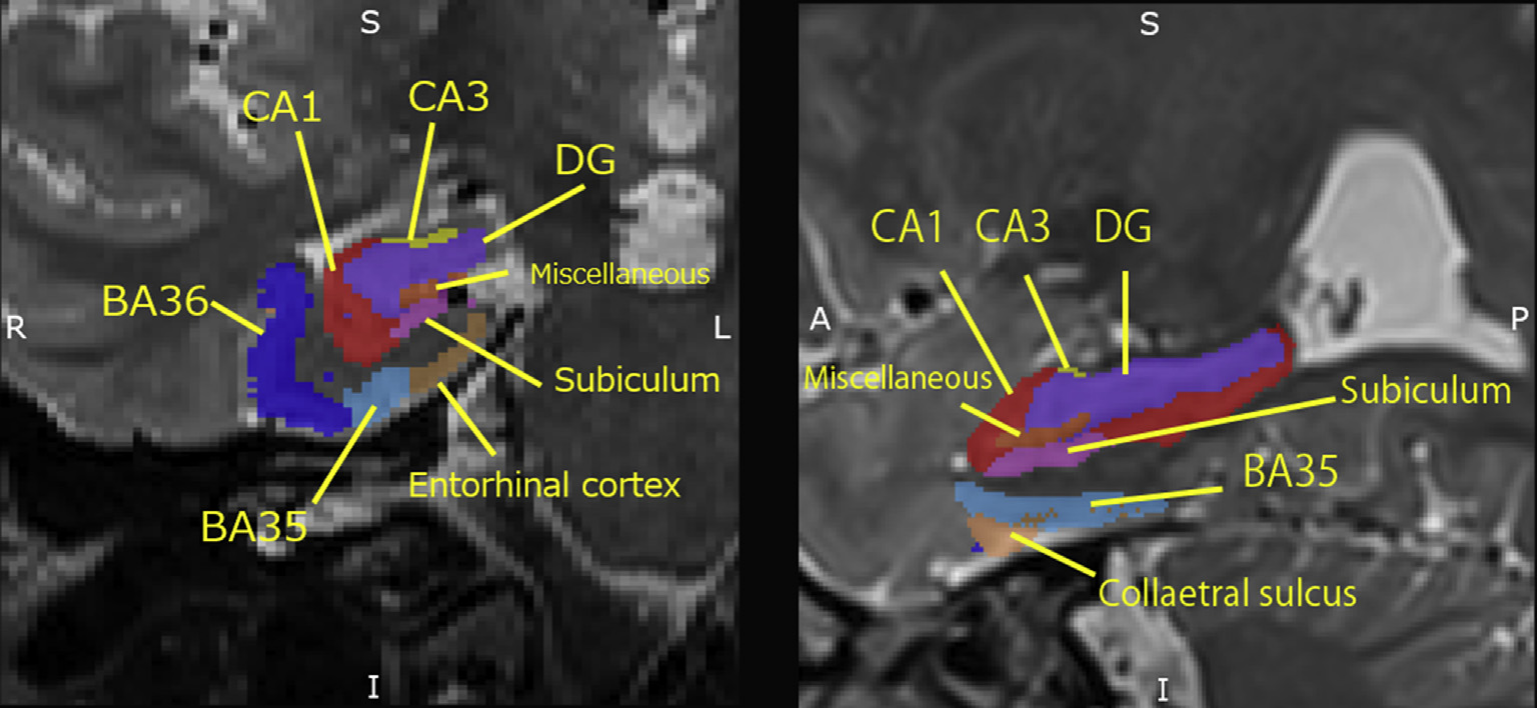
Automatically segmented subfield areas of the medial temporal lobe on high-resolution T2-weighted coronal (left) and sagittal (right) images.

Automatic segmentation of the medial temporal lobe is shown in Fig. 1. The subfield volumes of the medial temporal lobe are listed in Table 2. There were significant differences in the bilateral CA1, CA2, DG, subiculum, ERC, BA35, and BA36 among the three diagnostic groups (*p* < 0.001). All paired samples t-tests on the left versus right comparisons of volumetric data are presented in Table 3. There were significant differences in volumes between the left and the right CA1, CA2, CA3, DG, BA36,CS.

**Table 2:** Differences in the subfield volumes of the medial temporal lobe among the three groups.

Left subfield	Volume (mm^3^)			ANOVA		Right subfield	Volume (mm^3^)			ANOVA	
	HC (n = 50)	MCI (n = 14)	AD (n = 17)	F value	*p* value		HC (N = 50)	MCI (n = 14)	AD (n = 17)	F value	*p* value
CA1	1331.6 ± 166.9	1134.6 ± 266.1	958.4 ± 139.3	28.367	<0.001^∗∗^	CA1	1358.6 ± 149.4	1192.0 ± 265.8	952.2 ± 151.4	35.111	<0.001
CA2	21.7 ± 8.0	16.7 ± 6.5	12.6 ± 6.3	10.259	<0.001^∗∗^	CA2	25.4 ± 5.9	22.8 ± 6.2	18.1 ± 5.2	10.084	<0.001^∗∗^
CA3	71.2 ± 18.5	64.2 ± 15.1	68.6 ± 15.6	0.919	0.403	CA3	76.0 ± 19.7	75.2 ± 16.5	71.8 ± 17.0	0.321	0.726
DG	797.2 ± 102.7	672.2 ± 157.0	588.1 ± 78.4	25.633	<0.001^∗∗^	DG	826.3 ± 102.6	706.4 ± 158.5	592.3 ± 86.8	29.869	<0.001^∗∗^
MISC	160.7 ± 63.6	145.9 ± 29.9	177.5 ± 45.8	1.254	0.291	MISC	147.9 ± 47.2	154.3 ± 47.6	172.6 ± 30.3	1.973	0.146
Sub	404.0 ± 73.0	324.8 ± 82.8	303.0 ± 67.8	14.969	<0.001^∗∗^	Sub	398.6 ± 65.1	340.7 ± 76.8	290.9 ± 63.2	17.690	<0.001^∗∗^
ERC	519.1 ± 75.2	459.7 ± 122.1	391.3 ± 84.1	14.389	<0.001^∗∗^	ERC	522.0 ± 66.1	465.4 ± 107.7	344.5 ± 93.3	31.000	<0.001^∗∗^
BA35	435.5 ± 84.1	387.6 ± 98.5	331.5 ± 60.6	10.473	<0.001^∗∗^	BA35	442.0 ± 88.2	384.6 ± 116.4	305.2 ± 92.6	13.676	<0.001^∗∗^
BA36	1637.6 ± 330.1	1449.8 ± 339.1	1274.9 ± 214.4	9.148	<0.001^∗∗^	BA36	1528.5 ± 315.0	1376.7 ± 374.4	1128.5 ± 357.3	9.203	<0.001^∗∗^
CS	346.0 ± 145.4	327.7 ± 67.2	349.4 ± 152.5	0.116	0.890	CS	264.7 ± 147.7	201.8 ± 63.8	229.1 ± 113.1	1.464	0.238

**p < 0.001.

ANOVA, analysis of variance; HC, healthy control; MCI, mild cognitive impairment; AD, Alzheimer's disease.

CA1, Cornu ammonis 1; CA2, Cornu ammonis 2; CA3, Cornu ammonis 3; DG, dentate gyrus; MISC, miscellaneous; Sub, subiculum; ERC, entorhinal cortex; BA35, Brodmann area 35; BA36, Brodmann area 36; CS, collateral sulcus.

**Table 3:** Comparison of left and right subfield volumes with paired samples t-tests in all invdividuals..

	Left Mean	Right Mean	*t* value	*p* value
CA1 mm^3^	896	915	-2.17	0.033*
CA2 mm^3^	14	17	-4.53	<0.001*
CA3 mm^3^	113	119	-2.84	0.006*
DG mm^3^	476	490	-2.81	0.006*
MISC mm^3^	121	114	1.83	0.071
Subiculum mm^3^	272	270	0.58	0.566
ERC mm^3^	354	348	1.12	0.268
BA35 mm^3^	299	297	0.27	0.787
BA36 mm^3^	1126	1040	4.26	<0.001*
CS mm^3^	259	184	10.04	<0.001*

*p < 0.05.

CA1, Cornu ammonis 1; CA2, Cornu ammonis 2; CA3, Cornu ammonis 3.

DG, dentate gyrus; MISC, miscellaneous; ERC, entorhinal cortex; BA35, Brodmann area 35; BA36, Brodmann area 36; CS, collateral sulcus.

### 3.3 Associations between the subfield volumes of the medial temporal lobe and cognitive assessment scores

**Fig. 2:**
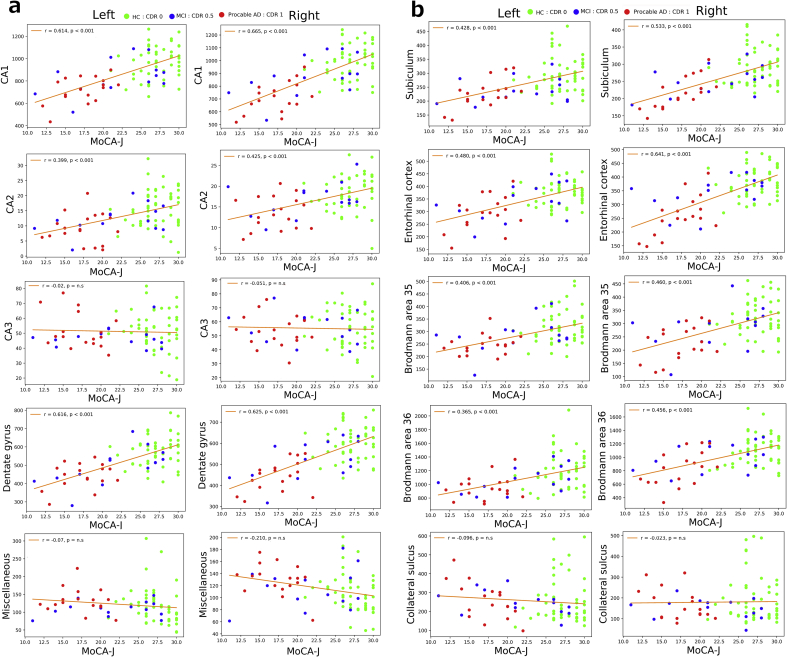
Correlations between the subfield volumes of the medial temporal lobe and MoCA-J scores in all individuals. Green, blue, and red dots show healthy control, MCI, and AD groups, respectively. All subfield volumes were adjusted by the total intracranial volume. a. Correlations between volumes of CA1,CA2,CA3, dentate gyrus, and miscellaneous, and MoCA-J scores. b. Correlations between volumes of subiculum, entorhinal cortex, Brodmann area 35, Brodmann area 36, and collateral sulcus, and MoCA-J scores.

For all individuals of the three diagnostic groups, significantly high correlation coefficients (above 0.6) were observed in five subfields (left CA1, left DG, right CA1, right DG, right ERC) for the MMSE and MoCA-J and in one subfield (right CA1) for the WMS-R LM II (*p* < 0.001; Table 4, Fig. 2). The MoCA-J and MMSE showed almost identical correlations in these subfields and higher correlation coefficients than did the WMS-R.

**Table 4:** Correlation coefficients of subfield volumes corrected for total intracranial volume with neuropsychological assessment scores in all individuals.

	left_ CA1	left_ CA2	left_ CA3	left_ DG	left_ MISC	left_ Sub	left_ ERC	left_ BA35	left_ BA36	left_ CS	right_ CA1	right_ CA2	right_ CA3	right_ DG	right_ MISC	right_ Sub	right_ ERC	right_ BA35	right_ BA36	right_ CS
MMSE	0.620**	0.426**	0.021	0.629**	0.031	0.441**	0.510**	0.450**	0.362*	-0.032	0.693**	0.407**	-0.018	0.654**	-0.088	0.562**	0.631**	0.497**	0.427**	0.066
MoCA-J	0.614**	0.399**	-0.020	0.616**	-0.072	0.428**	0.480**	0.406*	0.365*	-0.096	0.665**	0.425**	-0.051	0.626**	-0.210	0.533**	0.641**	0.460*	0.456**	0.023
WMS-RLM I	0.477**	0.345*	0.083	0.493**	0.016	0.378*	0.413**	0.410**	0.336*	-0.006	0.547**	0.334*	0.157	0.475**	-0.189	0.422**	0.555**	0.457**	0.372*	0.117
WMS-RLM II	0.531**	0.355*	0.052	0.533**	-0.044	0.399**	0.428**	0.381*	0.328*	-0.096	0.608**	0.398**	0.162	0.532**	-0.191	0.440**	0.579**	0.430*	0.363*	0.024

*p < 0.05, **p < 0.001.

CA1, Cornu ammonis 1; CA2, Cornu ammonis 2; CA3, Cornu ammonis 3; DG, dentate gyrus; MISC, miscellaneous; ERC, entorhinal cortex; sub, subiculum; BA35, Brodmann area 35; BA36, Brodmann area 36; CS, collateral sulcus; MMSE, Mini-Mental State Examination; MoCA-J, Montreal Cognitive Assessment Japanese Version; WMS-R LM I, Wechsler Memory Scale-revised Logical Memory I, WMS-R LM II; Wechsler Memory Scale-revised Logical Memory II.

For patients with MCI and AD, significantly high correlation coefficients (above 0.6) were observed in two subfields (right CA1 and right ERC) for the MMSE, in five subfields (left DG, right CA1, right DG, right subiculum, right ERC) for the MoCA-J, in one subfield (right CA1) for the WMS-R LM I, and in three subfields (left BA36, right CA1, right DG) for the WMS-R LM II (*p* < 0.001; Table 5). The MoCA-J showed higher correlation coefficients than did the MMSE in CA1, the DG, the subiculum, and the ERC.

**Table 5:** Correlation coefficients of subfield volumes corrected for total intracranial volume with neuropsychological assessment scores in patients with MCI and AD.

	left_ CA1	left_ CA2	left_CA3	left_ DG	left_MISC	left_ Sub	left_ ERC	left_ BA35	left_ BA36	left_ CS	right_ CA1	right_ CA2	right_CA3	right_DG	right_MISC	right_ Sub	right_ ERC	right_ BA35	right_ BA36	right_ CS
MMSE	0.533*	0.457*	-0.187	0.538*	-0.037	0.308	0.462*	0.503*	0.492*	-0.149	0.636**	0.372*	0.009	0.584*	-0.012	0.595*	0.605*	0.550*	0.486*	-0.072
MoCA-J	0.584*	0.355	-0.199	0.626**	-0.121	0.392*	0.559*	0.430*	0.520*	-0.450*	0.654**	0.428**	-0.120	0.637**	-0.058	0.625**	0.643**	0.491*	0.509**	-0.353
WMS-R LM I	0.478*	0.409*	-0.035	0.568*	-0.160	0.276	0.444*	0.455*	0.598*	-0.301	0.607**	0.418*	0.154	0.554*	-0.086	0.468*	0.473*	0.518*	0.439*	-0.202
WMS-R LM II	0.539*	0.325	-0.124	0.575*	-0.340	0.328	0.451*	0.316	0.612**	-0.434*	0.713**	0.477*	0.118	0.634*	-0.072	0.564*	0.544*	0.420*	0.472*	0.390

*p < 0.05, **p < 0.001.

CA1, Cornu ammonis 1; CA2, Cornu ammonis 2; CA3, Cornu ammonis 3; DG, dentate gyrus; MISC, miscellaneous; ERC, entorhinal cortex; sub, subiculum; BA35, Brodmann area 35; BA36, Brodmann area 36; CS, collateral sulcus; MMSE, Mini-Mental State Examination; MoCA-J, Montreal Cognitive Assessment Japanese Version; WMS-R LM I, Wechsler Memory Scale-revised Logical Memory I, WMS-R LM II; Wechsler Memory Scale-revised Logical Memory II.

## 4 Discussion

This study was conducted to clarify the subfields of the medial temporal lobe associated with cognitive assessment scores for the early diagnosis of AD. In partial correlation analysis with adjustment for age and sex, high correlation coefficients were found in cognitive assessment scores and the subfield volumes of CA1, the DG, the subiculum, the ERC, and BA36. In all healthy controls and individuals with MCI or AD, the MMSE and MoCA-J showed identical correlation coefficients in CA1, the DG, and the ERC and higher correlation coefficients in these subfields than the WMS-R. However, the MoCA-J showed higher correlation coefficients in CA1, the DG, the subiculum, and the ERC in patients with MCI or AD than did the MMSE.

Recent work revealed a prominent volume reduction in CA1 and the ERC in amnestic MCI [24]. Moreover, the CA1 volume showed a greater difference between MCI patients and healthy controls than the whole volume of the hippocampus. Mueller et al. [25] found an association between CA1 and a cognitive task assessing late retrieval and consolidation in individuals with subjective memory problems. The present findings of high correlations between the CA1 volume and cognitive assessment scores concurred well with this previous result.

High correlations between subfield volumes and MoCA-J were also observed in the DG, the subiculum, and the ERC in the present study. The DG is involved in memory encoding and early retrieval [26] and its volume is correlated with verbal memory and visuospatial memory in healthy controls [27]. The subiculum receives input from CA1 and the ERC and serves as the major output structure of the hippocampus. The subiculum has been associated with regulation of memory recall such as rapid memory updating [27, 28]. The ERC volume has been correlated with both delayed free recall and delayed recognition subtests [10, 29]. Information entering the DG of the hippocampus is transmitted from CA3 to CA1 and is sent to the ERC and frontal cortex [27]. Higher correlations between the subfield volumes and cognitive assessment scores in CA1, the DG, and ERC than for other areas may be related to this transfer process of information, which plays a role in memory writing and recall.

The reason why the MoCA-J showed higher correlation with these subfield volumes in MCI and AD groups than the MMSE may be greater inclusion of immediate recall, delayed recall, and visuospatial tasks in the MoCA-J than in the MMSE. The MoCA-J has 15 points with a total score of 30 in combination with 4 points for visuospatial abilities, 6 points for language abilities, and 5 points for short-term memory recall. However, the MMSE has only 8 points with a total score of 30 in combination with 1 point for visuospatial abilities, 4 points for language abilities, and 3 points for short-term memory recall. Another reason may be a lower ceiling effect from 28 to 30 points for MCI in the MoCA-J than in the MMSE [30]. Higher scores of the MMSE than the MoCA-J in the MCI group in the present study may be due to this ceiling effect. The MoCA-J may more quickly capture a decrease in subfield volumes in MCI than did the MMSE.

In the present study right CA1, CA2, CA3, DG were larger in volume than the same structures in the left hemisphere. This is in line with a previous study on hippocampal subfield volume measures in healthy subjects using FreeSurfer software program [31]. This previous study found that left CA1 volume significantly correlated with delayed recall of California Verbal Learning Test II. In contrast the present study showed the right CA1 volume significantly correlated with delayed recall of WMS-LMII. This left-right differences in correlation between CA1 volume and delayed recall performance may be attributed to differences in study cohorts, cognitive assessments, and used software programs.

This study has several limitations. First, the number of study patients with MCI and AD is rather small compared with healthy controls. Second, because this study was conducted for patients visiting our hospital and healthy volunteers living in the neighborhood, it is unclear whether the results can be generalized to elderly individuals living in other areas. Third, the present study investigated the relationship between subfield volumes and the total scores of cognitive assessments. Further clarification of the relationship between subfield volumes and subitems of cognitive assessments may be necessary.

## Declarations

### Author contribution statement

Masayo Ogawa: Conceived and designed the experiments; Performed the experiments; Analyzed and interpreted the data; Wrote the paper.

Daichi Sone, Iman Behesht, Hiroshi Matsuda: Analyzed and interpreted the data.

Norihide Maikusa, Kyoji Okita, Harumasa Takano: Contributed reagents, materials, analysis tools or data.

### Funding statement

This research did not receive any specific grant from funding agencies in the public, commercial, or not-for-profit sectors.

### Competing interest statement

The authors declare no conflict of interest.

### Additional information

No additional information is available for this paper.
